# Lead Optimization of 2-Cyclohexyl-*N*-[(*Z*)-(3-methoxyphenyl/3-hydroxyphenyl) methylidene]hydrazinecarbothioamides for Targeting the HER-2 Overexpressed Breast Cancer Cell Line SKBr-3

**DOI:** 10.3390/molecules201018246

**Published:** 2015-10-07

**Authors:** Mashooq A. Bhat, Abdullah Al-Dhfyan, Ahmed M. Naglah, Azmat Ali Khan, Mohamed A. Al-Omar

**Affiliations:** 1 Department of Pharmaceutical Chemistry, College of Pharmacy, King Saud University, P.O. Box 2457, Riyadh 11451, Saudi Arabia; E-Mail: azmatbiotech@gmail.com (A.A.K.); 2 Stem Cell & Tissue Re-Engineering Program, Research Center, King Faisal Specialized Hospital & Research Center, MBC-03, P. O. Box 3354, Riyadh 11211, Saudi Arabia; E-Mail: pharm101696@hotmail.com; 3 Department of Pharmacology and Toxicology, College of Pharmacy, King Saud University, Riyadh 11451, Saudi Arabia; 4 Department of Pharmaceutical Chemistry, Drug Exploration & Development Chair (DEDC), College of Pharmacy, King Saud University, Riyadh 11451, Saudi Arabia; E-Mails: amnaglah@gmail.com (A.M.N.); malomar1@KSU.EDU.SA (M.A.A.-O.); 5 Peptide Chemistry Department, Chemical Industries Research Division, National Research Centre, Dokki, Cairo 12622, Egypt

**Keywords:** thiosemicarbazones, HER-2, SKBr-3 cells, BT-474 cells, cancer stem cells

## Abstract

Lead derivatives of 2-cyclohexyl-*N*-[(*Z*)-(3-methoxyphenyl/3-hydroxyphenyl) methylidene]hydrazinecarbothioamides **1**–**18** were synthesized, characterized and evaluated *in vitro* against HER-2 overexpressed breast cancer cell line SKBr-3. All the compounds showed activity against HER-2 overexpressed SKBr-3 cells with IC_50_ = 17.44 ± 0.01 µM to 53.29 ± 0.33 µM. (2*Z*)-2-(3-Hydroxybenzylidene)-*N*-(3-methoxyphenyl)hydrazinecarbothioamide (**12**, IC_50_ = 17.44 ± 0.01 µM) was found to be most potent compound of this series targeting HER-2 overexpressed breast cancer cells compared to the standard drug 5-fluorouracil (5-FU) (IC_50_ = 38.58 ± 0.04 µM). Compound **12** inhibited the cellular proliferation via DNA degradation.

## 1. Introduction

Thiosemicarbazones constitute an important class of pharmacophore that has been explored by medicinal chemists owing to its wide range of biological activities, which include antibacterial, antimalarial, antiviral and antitumor activities [1,2,3]. Methisazone which was used for the treatment of smallpox, is an example of a thiosemicarbazone drug [4]. Thiosemicarbazone derivatives having potent anticancer activities have been reported in the literature [5,6,7,8]. Triapine, a potent anti-proliferative is effective against several cancer types. It obstructs tumor growth by inhibiting ribonucleotide reductase. Triapine (3-AP, 3-aminopyridine-2-carboxaldehyde thiosemicarbazone) has shown significant usefulness in the treatment of cancer and is currently in phase II clinical trials [9]. The therapeutic potential of 3-AP is however limited because of its poor water solubility and toxicity profile.

Worldwide the most common cancer and the second most leading cause of mortality in women is breast cancer, despite improvements in early detection. Recently the existence of breast cancer stem cells has been reported. Because of their resistance to conventional treatment, small populations of cells that are relatively resistant to therapy and able to repopulate *in vivo*, called cancer stem cells (CSCs), are believed to be responsible for treatment failures. CSCs have fundamental implications for the early detection, prevention and treatment of cancer. Drug discovery programs for cancer usually select compounds which have the property of inducing cytotoxic effects in cancer cell lines [10]. Unfortunately, the cytotoxic effects *in vitro* and inhibition of tumor growth *in vivo* is not the end story for curing cancer in preclinical models because of the existence of CSCs, as tumors are maintained by a self-renewing CSC population [11]. There are now various research findings which have confirmed cancer stem cells in leukemia [12], breast [13], brain [14], lung [15], colon [16]. To cause relapse, CSCs must have survived primary treatment [17]. Studies suggested that aldehyde dehydrogenase 1 (ALDH-1) is a more potent marker of breast CSCs [18,19,20]. ALDH-1-positive cells are resistant to conventional chemotherapy with paclitaxel and epirubicin [21]. ALDH-1-positive breast cancer CSCs can induce tumor formation with a few as 500 cells. Breast cancer cells that expressed ALDH-1 were more likely to be estrogen receptor (ER) negative, progesterone receptor (PR) negative and human-epidermal growth factor receptor-2 (HER-2) positive. Several reports demonstrated that HER-2 regulate CSCs. Cells displaying stem cell properties such as sphere formation or increased aldehyde dehydrogenase expression also have increased HER-2 expression compared with bulk cell population [22]. Traditionally breast cancer can be classified into three main subtypes: luminal, basal like and human epidermal growth factor receptor-2 positive (HER-2)^+^. Clinical and laboratory evidences have indicated that overexpression of HER-2 may render tumor cells resistant to several anticancer drugs [23]. Thus, there remains an urgent need for new pharmaceutical compounds and compositions to effectively eradicate and target cancer stem cells. We need to target both proliferating cells as well as cancer stem cells in order to cure cancer [24].

**Figure 1:**
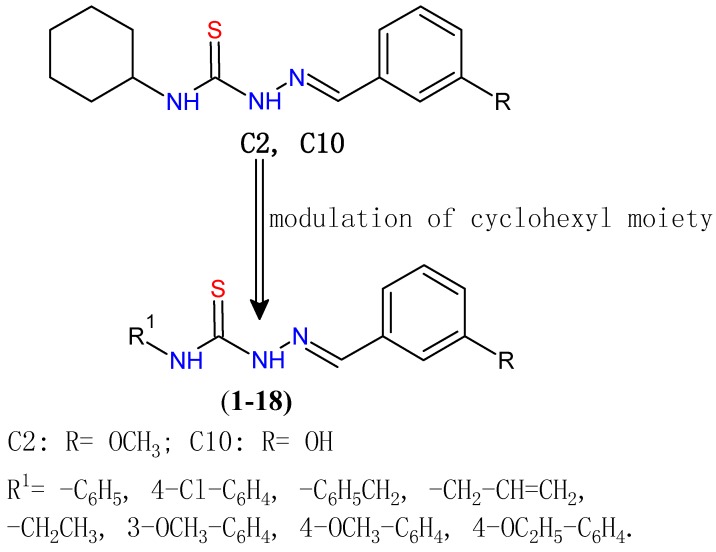
Structures of lead compound **C2**, **C10** and newly synthesized compounds **1**–**18**.

Therefore there is high potential in structural modification of thiosemicarbazone (TSC) derivatives to improve the existing drug candidates. In our previous research on TSC derivatives bearing a cyclohexyl moiety, the synthesized compounds showed activity against HER-2 expressed SKBr-3 cells with IC_50_ = 25.6 ± 0.07 µM − 61.6 ± 0.4 µM. The two compounds (2-cyclohexyl-*N*-[(*Z*)-(3-methoxyphenyl) methylidene]hydrazinecarbothioamide (**C2**) and (2-cyclohexyl-*N*-[(*Z*)-(3-hydroxyphenyl)- methylidene]hydrazinecarbothioamide (**C10**) (Figure 1) showed inhibitory effects on ALDH^+^ population more effectively than the reference drug, salinomycin. Compound **C2** was the most active, showing a 50% inhibitory effect [25]. These two compounds **C2** and **C10** were selected as lead compounds for further derivatization. Modulation of the cyclohexyl moiety was performed using different groups like phenyl, substituted phenyl, benzyl, alkyl, alkenyl, etc. In order to discover novel TSC derivatives with significant activity against CSCs. TCS derivatives bearing 3-hydroxy/3-methoxy phenyl on one side and different groups at the terminal nitrogen were synthesized and their antitumor activities on SKBr-3 and BT-474 cell line were determined. 

## 2. Results and Discussion

### 2.1. Chemistry

**Scheme 1:**
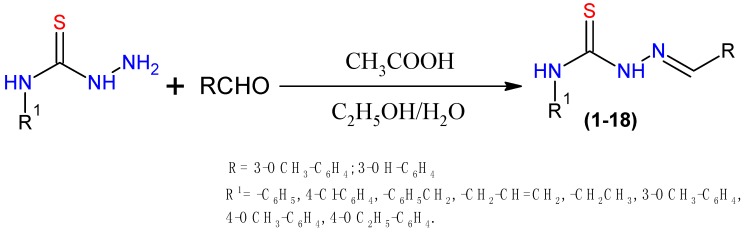
Synthetic protocol of compounds **1**–**18**.

The synthesis of thiosemicarbazone derivatives **1**–**18** was carried out in single step as shown in Scheme 1. Thiosemicarbazides were reacted with 3-methoxybenzaldehyde/3-hydroxybenzaldehyde in the presence of acetic acid to yield 2-cyclohexyl-*N*-[(*Z*)-(3-methoxyphenyl/3-hydroxyphenyl) methylidene]hydrazinecarbothioamide derivatives **1**–**18**. The purity of the compounds was checked by TLC and elemental analysis. The compounds were characterized and confirmed by spectral data. In the ^1^H-NMR spectra, the signals of the respective protons of the derivatives were verified on the basis of chemical shifts, multiplicity and coupling constants. The spectra of all synthesized compounds showed a D_2_O exchangeable singlet at 6.3–8.3 ppm and 11.1–11.7 ppm corresponding to NH protons and NHC=S protons. The analytical and spectral data of the compounds was in good agreement with the composition of the synthesized compounds. The physiochemical property data of the of all compounds is given in Table 1.

**Table 1:** Physical data of the synthesized compounds **1**–**18**.

Compounds	R	R^1^	Molecular Formula	Yield %	Mp (°C)	*C*Log P
**1**	3-Methoxyphenyl	Phenyl	C_15_H_15_N_3_OS	70	161–162	4.42
**2**	3-Methoxyphenyl	4-Chlorophenyl	C_15_H_14_ClN_3_OS	75	197–198	5.41
**3**	3-Methoxyphenyl	Prop-2-en	C_12_H_15_N_3_OS	72	115–116	2.70
**4**	3-Methoxyphenyl	Benzyl	C_16_H_17_N_3_OS	68	127–129	3.65
**5**	3-Methoxyphenyl	Ethyl	C_11_H_15_N_3_OS	72	122–123	2.40
**6**	3-Methoxyphenyl	3-Methoxyphenyl	C_16_H_17_N_3_O_2_S	65	148–150	4.68
**7**	3-Methoxyphenyl	4-Methoxyphenyl	C_16_H_17_N_3_O_2_S	70	156–157	4.37
**8**	3-Methoxyphenyl	4-Ethoxyphenyl	C_17_H_19_N_3_O_2_S	68	160–161	4.91
**9**	3-Methoxyphenyl	3-Chlorophenyl	C_15_H_14_ClN_3_OS	70	138–140	5.45
**10**	3-Hydroxyphenyl	Phenyl	C_14_H_13_N_3_OS	78	212–213	3.95
**11**	3-Hydroxyphenyl	4-Chlorophenyl	C_14_H_12_ClN_3_OS	65	222–224	4.94
**12**	3-Hydroxyphenyl	3-Methoxyphenyl	C_15_H_15_N_3_O_2_S	70	185–186	4.21
**13**	3-Hydroxyphenyl	Prop-2-en	C_11_H_13_N_3_OS	60	94–97	2.22
**14**	3-Hydroxyphenyl	Benzyl	C_15_H_15_N_3_OS	65	130–132	3.17
**15**	3-Hydroxyphenyl	4-Methoxyphenyl	C_15_H_15_N_3_O_2_S	72	182–183	3.90
**16**	3-Hydroxyphenyl	4-Ethoxyphenyl	C_16_H_17_N_3_O_2_S	70	200–202	4.43
**17**	3-Hydroxyphenyl	3-Chlorophenyl	C_14_H_12_ClN_3_OS	75	198–200	4.98
**18**	3-Hydroxyphenyl	Ethyl	C_10_H_13_N_3_OS	60	152–154	1.93

### 2.2. Anti-Proliferative *in Vitro* Activity

**Figure 2:**
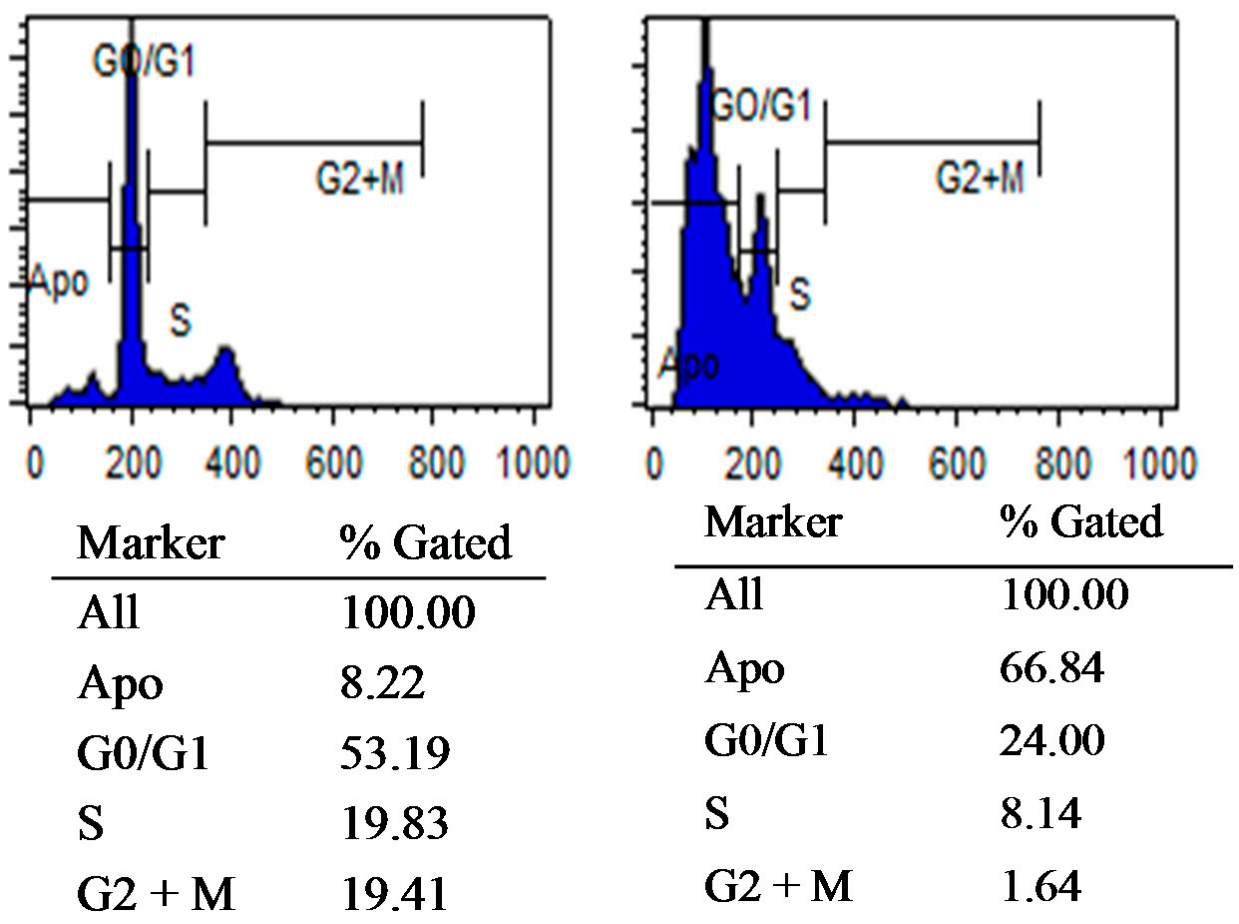
Cell cycle specific blocking activity of compound **12** on SKBr-3 cells.

*In vitro* anti-proliferative activity was measured by the cell growth inhibition assay. For the determination of IC_50_ for each compound, WST-1 reagent was used according to the protocol (Table 2). From our previous experience, TCS derivatives showed selectivity against HER-2 overexpressed cancer cells over luminal and basal subtypes. All the compounds showed activity against HER-2 overexpressed SKBr-3 cell with IC_50_ values ranging between 17.44 ± 0.01 µM to 53.29 ± 0.33 µM. Compound **12** (IC_50_ = 17.44 ± 0.01 µM) was found to be most potent compound of this series targeting HER-2 overexpressed breast cancer cells compared to the standard drug 5-fluorouracil (5-FU) (IC_50_ = 38.58 ± 0.04 µM). To gain insight into the anti-proliferation mechanism, the effect on cell cycle distribution was investigated by fluorescence-activated cell sorting (FACS) analysis. SKBr-3 cells were exposed to 10 µM of compound **12** for 48 h and the result was the accumulation of the cells on DNA degradation phase, which is a strong indication that the treatment induced apoptosis by breakdown of the cells’ DNA. This was also accompanied by a compensatory decrease in G_1_, S and M phase cells. Histograms show the number of cells per channel (vertical axis) *vs.* DNA content (horizontal axis). The values indicate the percentage of cells in the relevant phases of the cell cycle. The analysis shows increase in apoptosis of cells (DNA degradation) by 8 folds compared with untreated cells (Figure 2).

**Table 2:** *In vitro* cytotoxic activity of compounds against breast cancer cell line SKBr-3.

Compounds	^a^ IC_50_ (µM)
	SKBr-3
**1**	45.67 ± 0.18
**2**	23.69 ± 0.06
**3**	17.89 ± 0.03
**4**	34.52 ± 0.18
**5**	23.00 ± 0.16
**6**	24.55 ± 0.02
**7**	25.00 ± 0.03
**8**	23.76 ± 0.01
**9**	27.00 ± 0.03
**10**	22.32 ± 0.02
**11**	26.49 ± 0.02
**12**	17.44 ± 0.01
**13**	29.26 ± 0.05
**14**	27.74 ± 0.03
**15**	53.29 ± 0.33
**16**	24.75 ± 0.08
**17**	18.28 ± 0.05
**18**	22.33 ± 0.03
**5-FU**	38.58 ± 0.04

^a^ IC_50_: Concentration of the compound (μM) producing 50% cell growth inhibition after 48 h of compound exposure.

**Figure 3:**
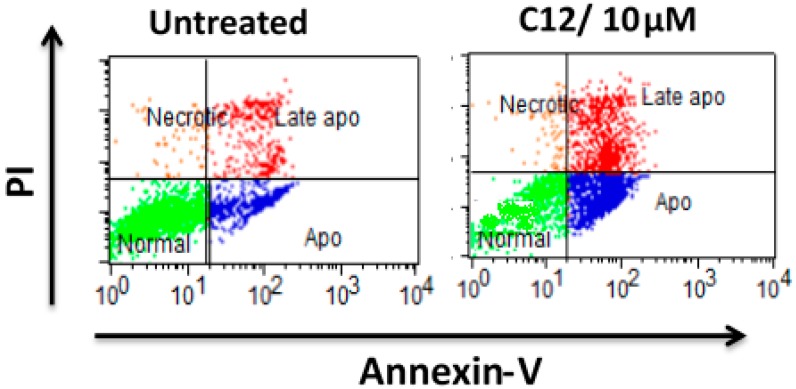
The apoptotic effect of compound **12** using Annexin-V staining of SKBr-3 cells.

These results suggested that compound **12** inhibited the cellular proliferation via DNA degradation. The apoptotic effect of compound **12** was evaluated using annexin-V staining. Cells were harvested after treatment by compound **12** (10 µM) for 48 h and incubated with annexin V-FITC and PI as described in the Experimental Section. Ten thousand cells were analyzed per determination. Dot plots show annexin V-FITC binding on the X axis and PI staining on the Y axis. Dots represent cells as follows: lower left quadrant, normal cells (FITC^−^/PI^−^); lower right quadrant, apoptotic cells (FITC^+^/PI^−^); upper left quadrant, necrotic cells (FITC^−^/PI^+^), upper right quadrant late apoptotic (FITC^+^/PI^+^) (Figure 3).

The apoptotic population increased from 16.3% in the control group to 49.14% and late apoptosis population increased from 10% to 26% increased after 48 h exposure time using 10 µM of compound **12** for incubation. The cytotoxic effect of compound was not correlated to the increase of necrotic cell population.

**Figure 4:**
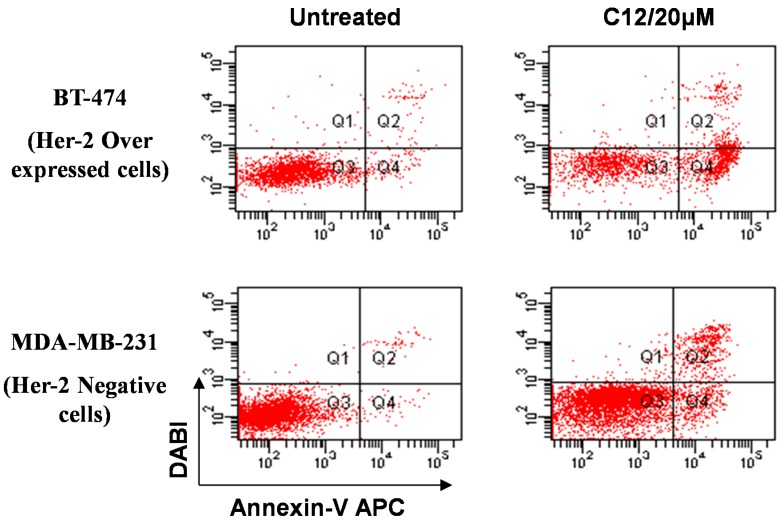
The apoptotic effect of compound **12** on HER-2 positive BT-474 and Her-2 negative MDA-MB-231 cells.

**Figure 5:**
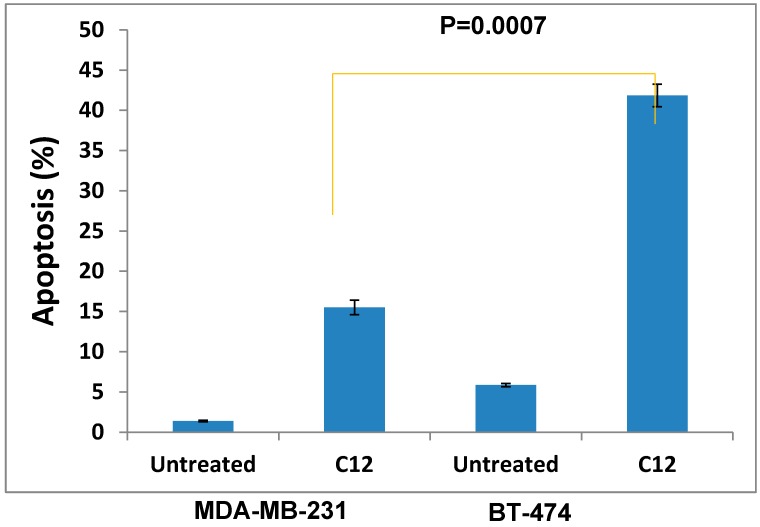
Histogram showing the % apoptosis of compound **12** on HER-2 negative MDA-MB-231 cells and HER-2 positive BT-474.

**Figure 6:**
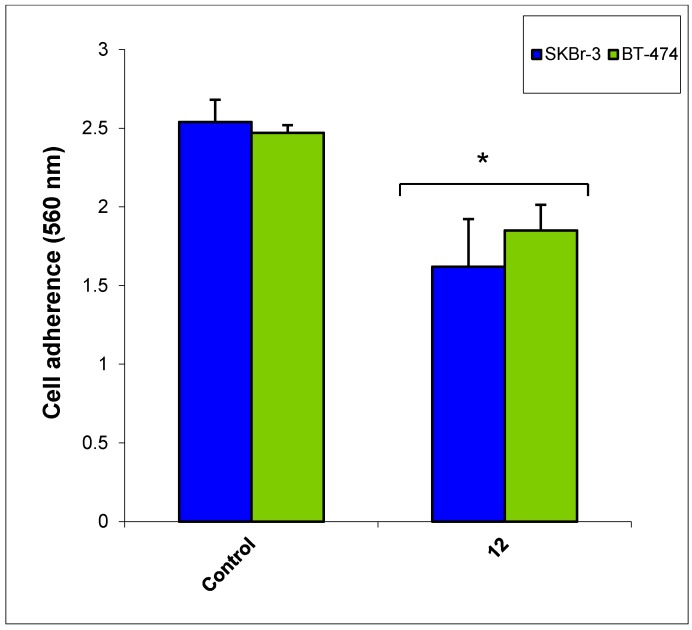
Effect of compound **12** on cell adherence of HER-2 positive cancer cell lines SKBr-3 and BT-474.

**Figure 7:**
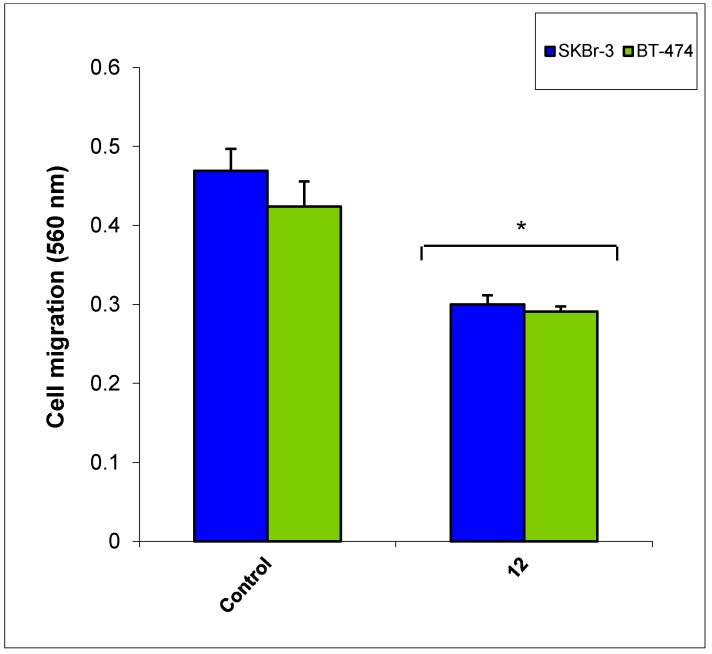
Effect of compound **12** on cell migration of HER-2 positive cancer cell lines SKBr-3 and BT-474.

**Figure 8:**
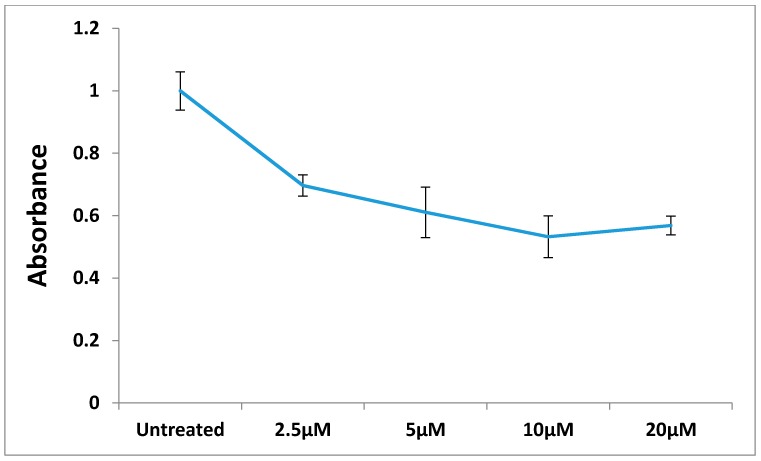
The absorbance of formazan dye produced by viable BT-474 cells treated with different concentrations of compound **12**.

**Figure 9:**
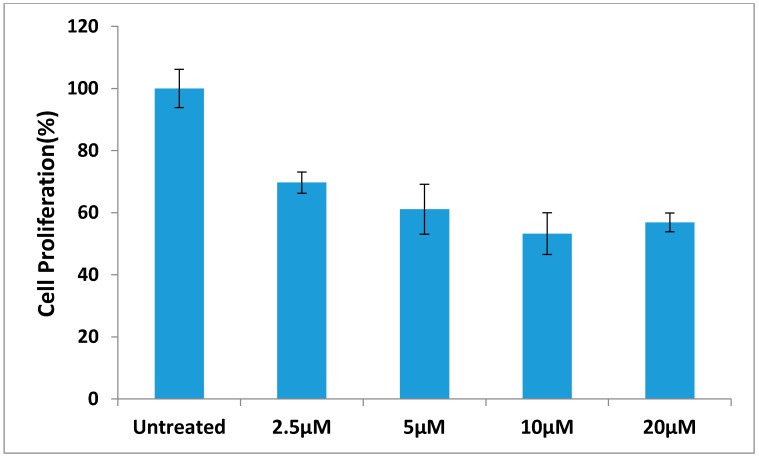
The percentage of viable/proliferative BT-474 cells treated with different concentrations of compound **12**.

The most active compound **12** presented apoptotic effects on overexpressed HER-2 oncogene and little sensitivity was measured on HER-2 negative MDA-MB-231 cells (Figure 4 and Figure 5). Cell adhesion inhibition studies were also carried out using the HER-2 positive SKBr-3 and BT-474 cancer cell lines. Compound **12** at tested concentrations significantly inhibited cell adhesion of SKBr-3 and BT-474 (*p* < 0.05), (Figure 6). The results shown in Figure 7 demonstrate that compound **12** had a maximum effect on cell migration of SKBr-3 and BT-474 cancer cells. It significantly inhibited cell migration of SKBr-3 and BT-474 (*p* < 0.05). Percentages of viable/proliferative BT-474 cells treated with different concentration of compound **12** were determined (Figure 8 and Figure 9). Cell proliferation inhibition was found to be significant at 10 µM concentration of compound **12**.

## 3. Experimental Section

### 3.1. General Information

All the solvents were obtained from Merck (Kenilworth, NJ, USA). The homogeneity of the compounds was checked by TLC performed on Silica gel G coated plates (Merck). An iodine chamber was used for visualization of TLC spots. The FT-IR spectra were recorded in KBr pellets on a Spectrum BX FT-IR spectrophotometer (Perkin Elmer, Hopkinton, MA, USA). The elemental analysis for C, H, N and S were within the limit of ±0.4% and ±0.3% of the theoretical values respectively. Melting points were determined on a Gallenkamp melting point apparatus, and the thermometer was uncorrected. NMR Spectra were scanned in DMSO-*d*_6_ on a Bruker NMR spectrophotometer operating at 500 MHz for ^1^H and 125.76 MHz for ^13^C. Chemical shifts δ are expressed in parts per million (ppm) relative to TMS as an internal standard and D_2_O was added to confirm the exchangeable protons. Coupling constants (*J*) are in hertz. The following abbreviations are used in the assignment of NMR signals: s (singlet), d (doublet), m (multiplet). Mass spectra were measured on triple quadruple mass spectroscopy (Waters, Corp. Milford, MA, USA).

#### 3.1.1. Representative Procedure for Synthesis of **1**–**18**

To a solution of thiosemicarbazide (0.0119 mol) in ethanol (11 mL), water (22 mL) was added. To this solution, 3-methoxybenzaldehyde/3-hydroxybenzaldehyde (0.0125 mol) and acetic acid (0.55 mL) were added. The mixture was stirred under reflux for 1 h and cooled to ambient temperature. After, the precipitate was collected with filter under vacuum and washed with water.

*(2Z)-2-(3-Methoxybenzylidene)-N-phenylhydrazinecarbothioamide* (**1**): Yield: 70%; 161–162; m.p.: 3143 °C; IR (KBr): (NH str.), 1578 (C=N str.), 1207 (NCSN str.), 1155 (C=S str.); ^1^H-NMR (DMSO-*d*_6_); δ = 3.8 (3H, s, -OCH_3_), 6.9–7.5 (9H, m, Ar-H), 8.1 (1H, s, N=CH), 10.1 (1H, s, NH, D_2_O exchg.), 11.8 (1H, s, NHCS, D_2_O exchg.); ^13^C-NMR (DMSO-*d*_6_): δ = 55.78 (OCH_3_), 112.36, 116.56, 121.20, 125.89, 126.59, 128.55, 130.20, 135.88, 139.58, 143.28, 160.05 (N=C), 176.58 (C=S); MS: *m*/*z* = 284.24 [M − 1]^+^; Analysis: for C_15_H_15_N_3_OS, calcd. C 63.13, H 5.30, N 14.73, S 11.24%; found C 63.25, H 5.20, N 14.53, S 11.21%.

*(2Z)-N-(4-Chlorophenyl)-2-(3-methoxybenzylidene) hydrazinecarbothioamide* (**2**): Yield: 75%; m.p.: 197–198 °C; IR (KBr): 3331 (NH str.), 1504 (C=N str.), 1273 (NCSN str.), 1155 (C=S str.); ^1^H-NMR (DMSO-*d*_6_); δ = 3.8 (3H, s, -OCH_3_), 7.0–7.6 (8H, m, Ar-H), 8.1 (1H, s, N=CH), 10.1 (1H, s, NH, D_2_O exchg.), 11.9 (1H, s, NHCS, D_2_O exchg.); ^13^C-NMR (DMSO-*d*_6_): δ = 55.78, 112.52, 116.56, 121.20, 128.16, 128.43, 129.88, 130.21, 131.20, 135.78, 138.56, 143.61, 160.04, 176.55. MS: *m*/*z* = 318.91 [M − 1]^+^; Analysis: for C_15_H_14_ClN_3_OS, calcd. C 56.33, H 4.41, N 13.14, S 10.03%; found C 56.12, H 4.40, N 13.12, S 10.00%.

*(2Z)-2-(3-Methoxybenzylidene)-N-(prop-2-en-1-yl)hydrazinecarbothioamide* (**3**): Yield: 72%; m.p.: 115–116 °C; IR (KBr): 3357 (NH str.), 1527 (C=N str.), 1286 (NCSN str.), 1156 (C=S str.); ^1^H-NMR (DMSO-*d*_6_); δ = 3.8 (3H, s, -OCH_3_), 4.2 (2H, d, -CH_2_), 5.1 (2H, m, CH_2_), 5.9 (1H, m, CH), 6.9–7.4 (4H, m, Ar-H), 8.0 (1H, s, N=CH), 8.7 (1H, s, NH, D_2_O exchg.), 11.5 (1H, s, NHCS, D_2_O exchg.); ^13^C-NMR (DMSO-*d*_6_): δ = 55.74, 112.46, 115.94, 116.05, 116.05, 120.67, 130.19, 135.58, 136.06, 142.44, 160.2, 177.74; MS: *m*/*z* = 251.00 [M − 2]^+^; Analysis: for C_12_H_15_N_3_OS, calcd. C 57.81, H 6.06, N 6.85, S 12.86%; found C 57.60, H 6.04, N 6.80, S 12.83%.

(*2Z)-N-Benzyl-2-(3-methoxybenzylidene)hydrazinecarbothioamide* (**4**): Yield: 68%; m.p.: 127–129 °C; IR (KBr): 3148 (NH str.), 1545 (C=N str.), 1254 (NCSN str.), 1041 (C=S str.); ^1^H-NMR (DMSO-*d*_6_); δ = 3.79 (3H, s, -OCH_3_), 4.8 (2H, s, -CH_2_), 6.9–7.4 (9H, m, Ar-H), 8.0 (1H, s, N=CH), 9.1 (1H, s, NH, D_2_O exchg.), 11.6 (1H, s, NHCS, D_2_O exchg.); ^13^C-NMR (DMSO-*d*_6_): δ = 47.05, 55.74, 112.55, 116.04, 120.04, 120.68, 127.17, 127.63, 128.63, 130.21, 136.04, 139.91, 142.62, 160.02, 178.14; MS: *m*/*z* = 300.09 [M + 1]^+^; Analysis: for C_16_H_17_N_3_OS, calcd. C 62.92, H 7.59, N 3.76, S 10.50%; found C 62.87, H 7.57, N 3.75, S 10.53%.

*(2Z)-N-Ethyl-2-(3-methoxybenzylidene)hydrazinecarbothioamide* (**5**): Yield: 72%; m.p.: 122–123 °C; IR (KBr): 3351 (NH str.), 1544 (C=N str.), 1272 (NCSN str.), 1153 (C=S str.); ^1^H-NMR (DMSO-*d*_6_); δ = 1.15 (3H, t, -CH_3_), 3.6 (2H, q, CH_2_), 3.8 (3H, s, -OCH_3_), 6.9–7.3 (4H, m, Ar-H), 8.0 (1H, s, N=CH), 8.5 (1H, s, NH, D_2_O exchg.), 11.4 (1H, s, NHCS, D_2_O exchg.); ^13^C-NMR (DMSO-*d*_6_): δ = 15.07, 55.72, 112.35, 116.05, 120.62, 130.21, 136.07, 142.22, 160.01, 177.16; MS: *m*/*z* = 237.07 [M]^+^; Analysis: for C_11_H_15_N_3_OS, calcd. C 55.67, H 6.37, N 17.71, S 13.51%; found C 55.87, H 6.35, N 17.73, S 13.48%.

*(2Z)-2-(3-Methoxybenzylidene)-N-(3-methoxyphenyl)hydrazinecarbothioamide* (**6**): Yield: 65%; m.p.: 148–150 °C; IR (KBr): 3308 (NH str.), 1599 (C=N str.), 1286 (NCSN str.), 1156 (C=S str.); ^1^H-NMR (DMSO-*d*_6_); δ = 3.8 (6H, s, -OCH_3_), 6.9–7.5 (8H, m, Ar-H), 8.1 (1H, s, N=CH), 10.0 (1H, s, NH, D_2_O exchg.), 11.8 (1H, s, NHCS, D_2_O exchg.); ^13^C-NMR (DMSO-*d*_6_): δ = 55.77, 104.15, 107.40, 110.66, 111.98, 112.44, 115.42, 116.55, 118.48, 121.17, 129.88, 130.69, 134.06, 135.83, 140.66, 141.79, 143.35, 148.55, 159.49, 160.11, 165.69, 176.31. MS: *m*/*z* = 315.44 [M]^+^; Analysis: for C_16_H_17_N_3_O_2_S, calcd. C 60.93, H 5.43, N 3.32, S 10.17%; found C 60.70, H 5.40, N 3.30, S 10.15%.

*(2Z)-2-(3-Methoxybenzylidene)-N-(4-methoxyphenyl)hydrazinecarbothioamide* (**7**): Yield: 70%; m.p.: 156–157 °C; IR (KBr): 3304 (NH str.), 1542 (C=N str.), 1236 (NCSN str.), 1152 (C=S str.); ^1^H-NMR (DMSO-*d*_6_); δ = 3.8 (6H, s, -OCH_3_), 6.9–7.5 (8H, m, Ar-H), 8.1 (1H, s, N=CH), 10.0 (1H, s, NH, D_2_O exchg.), 11.7 (1H, s, NHCS, D_2_O exchg.); ^13^C-NMR (DMSO-*d*_6_): δ = 55.69, 55.71, 55.78, 112.27, 113.76, 114.11, 116.50, 121.17, 126.58, 128.27, 130.18, 132.48, 132.71, 135.96, 143.03, 156.99, 157.51, 160.06, 176.98, 180.69; MS: *m*/*z* = 315.00 [M]^+^; Analysis: for C_16_H_17_N_3_O_2_S, calcd. C 60.93, H 5.43, N 13.32, S 10.17%; found C 60.71, H 5.42, N 13.34, S 10.15%.

*(2Z)-N-(4-Ethoxyphenyl)-2-(3-methoxybenzylidene)hydrazinecarbothioamide* (**8**): Yield: 68%; m.p.: 160–161 °C; IR (KBr): 3155 (NH str.), 1540 (C=N str.), 1274 (NCSN str.), 1115 (C=S str.); ^1^H-NMR (DMSO-*d*_6_); δ = 1.3 (3H, t, -CH_3_), 3.8 (3H, s, -OCH_3_), 4.0 (2H, q, -CH_2_), 6.9–7.5 (8H, m, Ar-H), 8.1 (1H, s, N=CH), 10.0 (1H, s, NH, D_2_O exchg.), 11.7 (1H, s, NHCS, D_2_O exchg.); ^13^C-NMR (DMSO-*d*_6_): δ = 15.16, 55.78, 63.63, 112.23, 114.23, 114.59, 116.51, 121.17, 128.24, 130.17, 132.35, 135.96, 142.99, 156.77, 160.05, 176.93; MS: *m*/*z* = 329.23 [M]^+^; Analysis: for C_17_H_19_N_3_O_2_S, calcd. C 61.98, H 5.81, N 12.76, S 9.73%; found C 61.75, H 5.84, N 12.74, S 9.75%.

*(2Z)-N-(3-Chlorophenyl)-2-(3-methoxybenzylidene)hydrazinecarbothioamide* (**9**): Yield: 70%; m.p.: 138–140 °C; IR (KBr): 3421 (NH str.), 1545 (C=N str.), 1269 (NCSN str.), 1152 (C=S str.); ^1^H-NMR (DMSO-*d*_6_); δ = 3.8 (3H, s, -OCH_3_), 7.0–7.7 (8H, m, Ar-H), 8.1 (1H, s, N=CH), 10.1 (1H, s, NH, D_2_O exchg.), 11.9 (1H, s, NHCS, D_2_O exchg.); ^13^C-NMR (DMSO-*d*_6_): δ = 55.78, 112.63, 116.57, 121.20, 124.88, 125.52, 125.84, 130.04, 130.21, 132.65, 135.74, 141.06, 143.80, 160.05, 176.40; MS: *m*/*z* = 319.00 [M]^+^; Analysis: for C_15_H_14_ClN_3_OS, calcd. C 56.33, H 4.41, N 13.14, S 10.03%; found C 56.50, H 4.40, N 13.15, S 10.00%.

*(2Z)-2-(3-Hydroxybenzylidene)-N-phenylhydrazinecarbothioamide* (**10**): Yield: 78%; m.p.: 212–213 °C; IR (KBr): 3311 (NH str.), 1542 (C=N str.), 1275 (NCSN str.), 1163 (C=S str.); ^1^H-NMR (DMSO-*d*_6_); δ = 6.8–7.6 (9H, m, Ar-H), 8.1 (1H, s, N=CH), 9.5 (1H, s, -OH, D_2_O exchg.), 10.1 (1H, s, NH, D_2_O exchg.), 11.7 (1H, s, NHCS, D_2_O exchg.); ^13^C-NMR (DMSO-*d*_6_): δ = 114.31, 117.78, 119.39, 125.74, 126.19, 128.31, 128.53, 128.75, 130.13, 135.76, 139.56, 143.69, 158.08, 176.43; MS: *m*/*z* = 265.30 [M − 6]^+^; Analysis: for C_14_H_13_N_3_OS, calcd. C 61.97, H 4.83, N 15.49, S 11.82%; found C 61.97, H 4.83, N 15.49, S 11.82%.

*(2Z)-N-(4-Chlorophenyl)-2-(3-hydroxybenzylidene)hydrazinecarbothioamide* (**11**): Yield: 65%; m.p.: 222–224 °C; IR (KBr): 3277 (NH str.), 1591 (C=N str.), 1276 (NCSN str.), 1196 (C=S str.); ^1^H-NMR (DMSO-*d*_6_); δ = 6.8–7.6 (9H, m, Ar-H), 8.0 (1H, s, N=CH), 9.5 (1H, s, -OH, D_2_O exchg.), 10.1 (1H, s, NH, D_2_O exchg.), 11.8 (1H, s, NHCS, D_2_O exchg.); ^13^C-NMR (DMSO-*d*_6_): δ = 114.35, 117.81, 119.41, 127.86, 128.39, 129.70, 130.11, 135.66, 138.57, 144.00, 158.07, 176.41; MS: *m*/*z* = 304.13 [M − 1]^+^; Analysis: for C_14_H_12_ClN_3_OS, calcd. C 54.99, H 3.96, N 13.74, S 10.49%; found C 54.78, H 3.95, N 13.73, S 10.47%.

*(2Z)-2-(3-Hydroxybenzylidene)-N-(3-methoxyphenyl)hydrazinecarbothioamide* (**12**): Yield: 70%; m.p.: 185–186 °C; IR (KBr): 3312 (NH str.), 1546 (C=N str.), 1277 (NCSN str.), 1155 (C=S str.); ^1^H-NMR (DMSO-*d*_6_); δ = 3.8 (6H, s, 2×-OCH_3_), 6.5–7.5 (8H, m, Ar-H), 8.1 (1H, s, N=CH), 9.6 (1H, s, -OH, D_2_O exchg.), 10.0 (1H, s, NH, D_2_O exchg.), 11.8 (1H, s, NHCS, D_2_O exchg.); ^13^C-NMR (DMSO-*d*_6_): δ = 55.62, 111.16, 11.58, 114.29, 115.11, 117.81, 118.08, 119.41, 121.59, 129.22, 130.14, 130.77, 135.71, 138.13, 140.65, 143.76, 158.08, 159.48, 176.14; MS: *m*/*z* = 302.30 [M + 1]^+^; Analysis: for C_15_H_15_N_3_O_2_S, calcd. C 59.78, H 5.02, N 13.94, S 10.64%; found C 59.57, H 5.00, N 13.96, S 10.62%.

*(2Z)-2-(3-Hydroxybenzylidene)-N-(prop-2-en-1-yl)hydrazinecarbothioamide* (**13**): Yield: 60%; m.p.: 94–97 °C; IR (KBr): 3357 (NH str.), 1585 (C=N str.), 1222 (NCSN str.), 1144 (C=S str.); ^1^H-NMR (DMSO-*d*_6_); δ = 4.2 (2H, d, -CH_2_), 5.1 (2H, m, =CH_2_), 5.9 (1H, m, =CH), 6.9–7.2 (4H, m, Ar-H), 7.9 (1H, s, N=CH), 8.6 (1H, s, -OH, D_2_O exchg.), 9.6 (1H, s, NH, D_2_O exchg.), 11.4 (1H, s, NHCS, D_2_O exchg.); ^13^C-NMR (DMSO-*d*_6_): δ = 46.19, 113.80, 116.03, 117.60, 119.14, 130.17, 135.42, 135.85, 142.98, 157.97, 177.59; MS: *m*/*z* = 235.12 [M]^+^; Analysis: for C_11_H_13_N_3_OS, calcd. C 56.15, H 5.57, N 17.86, S 13.63%; found C 56.26, H 5.55, N 17.87, S 13.60%.

*(2Z)-N-Benzyl-2-(3-hydroxybenzylidene)hydrazinecarbothioamide* (**14**): Yield: 65%; m.p.: 130–132 °C; IR (KBr): 3360 (NH str.), 1551 (C=N str.), 1301 (NCSN str.), 1147 (C=S str.); ^1^H-NMR (DMSO-*d*_6_); δ = 4.8 (2H, s, -CH_2_), 6.8–7.3 (9H, m, Ar-H), 8.0 (1H, s, N=CH), 9.0 (1H, s, -OH, D_2_O exchg.), 9.6 (1H, s, NH, D_2_O exchg.), 11.5 (1H, s, NHCS, D_2_O exchg.); ^13^C-NMR (DMSO-*d*_6_): δ = 18.99, 47.05, 56.55, 114.00, 117.58, 119.10, 127.20, 127.67, 128.64, 130.13, 135.90, 139.90, 143.09, 158.03, 177.99; MS: *m*/*z* = 285 [M]^+^; Analysis: for C_15_H_15_N_3_OS, calcd. C 63.13, H 5.30, N 4.73, S 11.24%; found C 63.36, H 5.32, N 4.75, S 11.22%.

*(2Z)-2-(3-Hydroxybenzylidene)-N-(4-methoxyphenyl)hydrazinecarbothioamide* (**15**): Yield: 72%; m.p.: 182–183 °C; IR (KBr): 3169 (NH str.), 1581 (C=N str.), 1283 (NCSN str.), 1022 (C=S str.); ^1^H-NMR (DMSO-*d*_6_); δ = 3.7 (6H, s, 2× -OCH_3_), 6.8–7.4 (8H, m, Ar-H), 8.0 (1H, s, N=CH), 9.5 (1H, s, -OH, D_2_O exchg.), 9.9 (1H, s, NH, D_2_O exchg.), 11.7 (1H, s, NHCS, D_2_O exchg.); ^13^C-NMR (DMSO-*d*_6_): δ = 55.69, 113.72, 114.11, 114.28, 114.66, 117.66, 118.86, 119.31, 126.58, 127.96, 130.09, 132.09, 132.45, 132.70, 135.84, 143.39, 156.99, 157.39, 158.06, 176.79, 180.67; MS: *m*/*z* = 302.49 [M + 1]^+^; Analysis: for C_15_H_15_N_3_O_2_S, calcd. C 59.78, H 5.02, N 13.94, S 10.64%; found C 59.55, H 5.00, N 13.92, S 10.66%.

*(2Z)-N-(3-Methoxyphenyl)-2-(3-hydroxybenzylidene)hydrazinecarbothioamide* (**16**): Yield: 70%; m.p.: 200–202 °C; IR (KBr): 3138 (NH str.), 1510 (C=N str.), 1280 (NCSN str.), 1039 (C=S str.); ^1^H-NMR (DMSO-*d*_6_); δ = 3.8 (3H, s, -OCH_3_), 4.0 (2H, q, -CH_2_), 6.9–7.5 (8H, m, Ar-H), 8.1 (1H, s, N=CH), 9.5 (1H, s, -OH, D_2_O exchg.), 10.0 (1H, s, NH, D_2_O exchg.), 11.7 (1H, s, NHCS, D_2_O exchg.); ^13^C-NMR (DMSO-*d*_6_): δ = 15.16, 55.78, 112.23, 114.23, 114.59, 116.51, 121.17, 128.24, 130.17, 132.35, 135.96, 142.99, 156.77, 160.05, 176.93; MS: *m*/*z* = 315.10 [M]^+^; Analysis: for C_16_H_17_N_3_O_2_S, calcd. C 60.93, H 5.43, N 13.32, S 10.17%; found C 60.70, H 5.41, N 13.30, S 10.14%.

*(2Z)-N-(3-Chlorophenyl)-2-(3-hydroxybenzylidene)hydrazinecarbothioamide* (**17**): Yield: 75%; m.p.: 198–200 °C; IR (KBr): 3281 (NH str.), 1589 (C=N str.), 1276 (NCSN str.), 1172 (C=S str.); ^1^H-NMR (DMSO-*d*_6_); δ = 6.8–7.7 (8H, m, Ar-H), 8.1 (1H, s, N=CH), 9.5 (1H, s, -OH, D_2_O exchg.), 10.1 (1H, s, NH, D_2_O exchg.), 11.9 (1H, s, NHCS, D_2_O exchg.); ^13^C-NMR (DMSO-*d*_6_): δ = 114.38, 117.88, 119.46, 124.56, 125.37, 125.54, 130.02, 130.12, 132.60, 135.61, 141.06, 144.18, 158.07, 176.24; MS: *m*/*z* = 304.58 [M − 1]^+^; Analysis: for C_14_H_12_ClN_3_OS, calcd. C 54.99, H 3.96, N 13.74, S 10.49%; found C 54.76, H 3.94, N 13.76, S 10.46%.

*(2Z)-N-Ethyl-2-(3-hydroxybenzylidene)hydrazinecarbothioamide* (**18**): Yield: 60%; m.p.: 152–154 °C; IR (KBr): 3358 (NH str.), 1557 (C=N str.), 1239 (NCSN str.), 1168 (C=S str.); ^1^H-NMR (DMSO-*d*_6_); δ = 1.15 (3H, t, CH_3_), 3.6 (2H, q, CH_2_), 6.8–7.2 (4H, m, Ar-H), 7.4 (1H, s, N=CH), 8.4 (1H, s, NH, D_2_O exchg.), 9.5 (1H, s, -OH, D_2_O exchg.), 11.3 (1H, s, NHCS, D_2_O exchg.); ^13^C-NMR (DMSO-*d*_6_): δ = 15.07, 113.96, 117.46, 118.95, 130.09, 135.97, 142.60, 158.04, 177.10; MS: *m*/*z* = 223.18 [M]^+^; Analysis: for C_10_H_13_N_3_OS, calcd. C 53.79, H 5.87, N 8.82, S 14.36%; found C 53.58, H 5.85, N 8.84, S 14.34%.

### 3.2. Cell Lines

SKBr-3, BT-474 and MDA-MB-231 breast cancer cell lines were purchased from the American Type Culture Collection (0801 University Boulevard, Manassas, VA, USA). SKBR-3 cells were cultured in McCoy’s 5A (GIBCO, 8717, Grovement Cir, Gaitherberg, MD, USA), and BT-474, MDA-MB-231 cells were cultured in DMEM (Sigma, 82024 Taufkirchen, Germany). The media supplemented with 10% FBS (Cambrex Bio Science, Baltimore, MD, USA), 100 IU/mL penicillin and 100 mg/mL streptomycin. Cell viability was assessed by trypan blue exclusion analysis. Cell numbers were determined by using a hemacytometer.

#### 3.2.1. WST-1 Cell Proliferation Assay

Cells were seeded into 96-well plates at 0.4 × 10^4^/well and incubated overnight. The medium was replaced with fresh one containing the desired concentrations of the compounds. After 48 h, 10 μL of the WST-1 reagent was added to each well and the plates were re-incubated for 4 h at 37 °C. The amount of formazan was quantified using an ELISA reader at 450 nm.

#### 3.2.2. Measurement of IC_50_

Cells were seeded into 96-well plates at 0.4 × 10^4^/well and incubated overnight. The medium was replaced with fresh one containing the desired concentrations of the compounds. After 48 h, 10 μL of the WST-1 reagent was added to each well and the plates were re-incubated for 4 h at 37 °C. The amount of formazan was quantified using an ELISA reader at 450 nm. For the compounds and the reference chemotherapeutic agent 5-FU, cells were cultured one day before treatment. Fresh media with fixed dose of 20 µM were replaced. IC_50_ was mathematically calculated as IC_50_ = fixed dose (20) × 50/(formazan quantity of treated cells/formazan quantity of untreated cells) × 100.

#### 3.2.3. Flow Cytometric Analysis of Cellular DNA Content

2 × 10^6^ cells were fixed in 1 mL ethanol (70%) for 60 min at room temperature. Harvested cells were re-suspended in 1 mL Na citrate (50 mM) containing 250 μg RNase A and incubated at 50 °C for 60 min. Next, cells were re-suspended in the same buffer containing 4 μg propidium iodine (PI) and incubated for 30 min before being analyzed by flow cytometry (Becton Dickinson, San Jose, CA, USA). The percentage of cells in various cell cycle phases was determined by using Cell Quest Pro software (Becton Dickinson).

#### 3.2.4. Measurement of Annexin-V Binding by Flow Cytometry

It has been shown that loss of phospholipid asymmetry of the plasma membrane is an early event of apoptosis. The annexin-V binds to negatively charged phospholipids, like phosphatidylserine. During apoptosis, the cells react to annexin-V once chromatin condenses but before the plasma membrane loses its ability to exclude propidium iodide (PI). Hence, by staining cells with a combination of fluorescein isothiocyanate (FITC) annexin-V and PI, it is possible to detect non-apoptotic live cells, early apoptotic cells and late apoptotic or necrotic cells. Annexin-V staining was performed by using Vybrant Apoptosis Assay Kit # 2 (Molecular Probe, Eugene, Oregon, 97402-0469) following the manufacturer’s recommendations. Annexin-V stained cells were analyzed by flow cytometry, measuring the fluorescence emission at 530 and less than 575 nm.

#### 3.2.5. Cancer Cell Migration Assay

Cell migration assay was performed according to the standard protocol. Three concentration 25 µM of compound was taken for testing. The lower well of the migration plate was supplemented with 500 μL of DMEM containing 10% fetal bovine serum with or without (vehicle control ethanol only) test compound. To the inside of each insert 100 μL of 0.5–1.0 × 10^6^ cells/mL of SKBr-3/BT-474 cell suspension was added separately. The plates were incubated for 8 h at 37 °C in a humidified CO_2_ incubator. After incubation, the media from inside of the inserts was carefully aspirated and the non-migratory cells were removed using cotton-tipped swabs. The inserts were transferred to a clean well containing 400 μL of cell stain solution and incubated for 10 min at room temperature. The stained inserts were gently washed several times and then transferred again to an empty well. Finally, 200 μL of extraction solution per well was added and incubated for 10 min on an orbital shaker. From each sample, 100 μL was taken in a 96-well microtiter plate and the absorbance at 560 nm was read in a plate reader.

#### 3.2.6. Cancer Cell Adhesion Assay

Under sterile conditions, the adhesion plate was allowed to warm up at room temperature for 10 min. 150 μL of 0.1–1.0 × 10^6^ cells/mL of SKBr-3/BT-474 cell suspension in serum free media with vehicle control ethanol only or test compound (25 µM) was added to the inside of each well. The plates were incubated for 30–90 min in a CO_2_ incubator. The wells were washed three times with PBS and the adhered cells were stained with 200 μL of cell stain solution for 10 min at room temperature. The excess stain was removed by washing 4–5 times with distilled water. After air drying the wells, 200 μL of extraction solution per well was added and then incubated for 10 min on an orbital shaker. The 150 μL from each extracted sample was transferred to a 96-well microtiter plate and the absorbance at 560 nm was read in a plate reader. Absorbance of dye in the control (vehicle-treated) cells was regarded as 100% adherence and the percentage adherence of treated cells was calculated in comparison with that of the control cells. Cell Migration and Cell Adhesion Assay kits were obtained from Cell Biolabs, Inc. (San Diego, CA, USA).

#### 3.2.7. The Effect of Different Concentration of Compound **12** on BT-474 Cells Proliferation

The cytotoxic effects of the compound **12** on BT-474 cell line was assayed by the MTT assay. The cells were seeded at a density of 5 × 10^4^ cells/well. The compound was serially diluted to final concentration of 20 µM, 10 µM, 5 µM and 2.5 µM. 200 μL liquid of each concentration was applied to the wells of a 96-well plate containing confluent cell monolayers (six wells per concentration). The dilution medium without the sample served as a control. After 48 h of incubation, MTT solution (5 mg/mL) was then added to each well, and the formazan precipitate was dissolved in 200 μL dimethyl sulfoxide after 4 h incubation. The content of the wells was homogenized on a microplate shaker for 5 min. The optical densities (OD) were measured on a microplate ELISA reader at 492 nm. All tests and analyses were run in triplicate and mean values were recorded. The cell survival curves were calculated after comparing with the control. The percentage viability was calculated as follows:

%viability = mean absorbance of treated wells × 100 mean absorbance of untreated wells

## 4. Conclusions

In conclusion, we focused on the design and synthesis of 2-cyclohexyl-*N*-[(*Z*)-(3-methoxyphenyl/3-hydroxyphenyl)methylidene]hydrazinecarbothioamides **1**–**18**, which were fully characterized by spectral analysis. The synthesized compounds were screened *in vitro* against HER-2 overexpressed breast cancer cell lines SKBr-3. Compound **12** presented the most significant activity against HER-2 over expressed breast cancer cell lines SKBr-3 and BT-474. Compound **12** significantly inhibited the cell migration and cell adhesion of breast cancer cell lines. Compound **12** was found to most active compound of this series and represents a good lead for development of drugs, targeting HER-2 over- expressed breast cancer cell lines.

## References

[1] Pelosi G. (2010). Thiosemicarbazone metal complexes: from structure to activity. Open. Crystallogr. J..

[2] Dilović I., Rubcić M., Vrdoljak V., Pavelić S.K., Kralj M., Piantanida I., Cindrić M. (2008). Novel thiosemicarbazone derivatives as potential antitumor agents: Synthesis, physicochemical and structural properties, DNA interactions and antiproliferative activity. Bioorg. Med. Chem..

[3] Kovacevic Z., Chikhani S., Lui G.Y., Sivagurunathan S., Richardson D.R. (2013). The iron-regulated metastasis suppressor NDRG1 targets NEDD4L, PTEN, and SMAD4 and inhibits the PI3K and Ras signaling pathways. Antioxid. Redox Signal..

[4] Heiner G.G., Fatima N., Russell P.K., Haase A.T., Ahmad N., Mohammed N., Thomas D.B., Mack T.M., Khan M.M., Knatterud G.L. (1971). Field trials of methisazone as a prophylactic agent against smallpox. Am. J. Epidemiol..

[5] Jutten P., Schumann W., Hartl A., Dahse H.M., Grafe U. (2007). Thiosemicarbazones of formyl benzoic acids as novel potent inhibitors of estrone sulfatase. J. Med. Chem..

[6] Yogeeswari P., Sriram D., Thirumurugan R., Raghavendran J.V., Sudhan K., Pavana R.K., Stables J. (2005). Discovery of *N*-(2,6-dimethylphenyl)-substituted semicarbazones as anticonvulsants: Hybrid pharmacophore-based design. J. Med. Chem..

[7] Greenbaum D.C., Mackey Z., Hansell E., Doyle P., Gut J., Caffrey C.R., Lehrman J., Rosenthal P.J., McKerrow J.H., Chibale K. (2004). Synthesis and structure-activity relationships of parasiticidal thiosemicarbazone cysteine protease inhibitors against Plasmodium falciparum, Trypanosoma brucei, and Trypanosoma cruzi. J. Med. Chem..

[8] Neve R.M., Chin K., Fridlyand J., Yeh J., Baehner F.L., Fevr T., Clark L., Bayani N., Coppe J.P., Tong F. (2006). A collection of breast cancer cell lines for the study of functionally distinct cancer subtypes. Cancer Cell.

[9] Finch R.A., Liu M., Grill S.P., Rose W.C., Loomis R., Vasquez K.M., Cheng Y., Sartorelli A.C. (2000). Triapine (3-aminopyridine-2-carboxaldehyde- thiosemicarbazone): A potent inhibitor of ribonucleotide reductase activity with broad spectrum antitumor activity. Biochem. Pharmacol..

[10] Winquist R.J., Furey B.F., Boucher D.M. (2010). Cancer stem cells as the relevant biomass for drug discovery. Curr. Opin. Pharmacol..

[11] McDermott S.P., Wicha M.S. (2010). Targeting breast cancer stem cells. Mol. Oncol..

[12] Bonnet D., Dick J.E. (1997). Human acute myeloid leukemia is organized as a hierarchy that originates from a primitive hematopoietic cell. Nat. Med..

[13] Al-Hajj M., Wicha M.S., Benito-Hernandez A., Morrison S.J., Clarke M.F. (2003). Prospective identification of tumorigenic breast cancer cells. Proc. Natl. Acad. Sci. USA.

[14] Singh S.K., Clarke I.D., Terasaki M., Bonn V.E., Hawkins C., Squire J., Dirks P.B. (2003). Identification of a cancer stem cell in human brain tumors. Cancer Res..

[15] Ho M.M., Ng A.V., Lam S., Hung J.Y. (2007). Side population in human lung cancer cell lines and tumors is enriched with stem-like cancer cells. Cancer Res..

[16] Ricci-Vitiani L., Lombardi D.G., Pilozzi E., Biffoni M., Todaro M., Peschle C., de Maria R. (2007). Identification and expansion of human colon-cancer-initiating cells. Nature.

[17] Bomken S., Fiser K., Heidenreich O., Vormoor J. (2010). Understanding the cancer stem cell. Br. J. Cancer.

[18] Ginestier C., Hur M.H., Charafe-Jauffret E., Monville F., Dutcher J., Brown M., Jacquemier J., Viens P., Kleer C.G., Liu S. (2007). ALDH1 is a marker of normal and malignant human mammary stem cells and a predictor of poor clinical outcome. Cell Stem Cell.

[19] Morimoto K., Kim S.J., Tanei T., Shimazu K., Tanji Y., Taguchi T., Tamaki Y., Terada N., Noguchi S. (2009). Stem cell marker aldehyde dehydrogenase 1-positive breast cancers are characterized by negative estrogen receptor, positive human epidermal growth factor receptor type 2, and high Ki67 expression. Cancer Sci..

[20] Charafe-Jauffret E., Ginestier C., Iovino F., Tarpin C., Diebel M., Esterni B., Houvenaeghel G., Extra J.M., Bertucci F., Jacquemier J. (2010). Aldehyde dehydrogenase 1-positive cancer stem cells mediate metastasis and poor clinical outcome in inflammatory breast cancer. Clin. Cancer Res..

[21] Tanei T., Morimoto K., Shimazu K., Kim S.J., Tanji Y., Taguchi T., Tamaki Y., Noguchi S. (2009). Association of breast cancer stem cells identified by aldehyde dehydrogenase 1 expression with resistance to sequential Paclitaxel and epirubicin-based chemotherapy for breast cancers. Clin. Cancer Res..

[22] Magnifico A., Albano L., Campaner S., Delia D., Castiglioni F., Gasparini P., Sozzi G., Fontanella E., Menard S., Tagliabue E. (2009). Tumor-initiating cells of HER2-positive carcinoma cell lines express the highest oncoprotein levels and are sensitive to trastuzumab. Clin. Cancer Res..

[23] Knuefermann C., Lu Y., Liu B., Jin W., Liang K., Wu L., Schmidt M., Mills G.B., Mendelsohn J., Fan Z. (2003). HER2/PI-3K/Akt activation leads to a multidrug resistance in human breast adenocarcinoma cells. Oncogene.

[24] Compton C.C. (2003). Colorectal carcinoma: diagnostic, prognostic, and molecular features. Mod. Pathol..

[25] Bhat M.A., Al-Dhfyan A., Khan A.A., Al-Harbi N., Manogaran P.S., Alanazi A.M., Fun H.K., Al-Omar M.A. (2015). Targeting HER-2 over expressed breast cancer cells with 2-cyclohexyl-*N*-[(*Z*)-(substituted phenyl/furan-2-yl/thiophene-2-yl)methylidene]hydrazinecarbothioamide. Bioorg. Med. Chem. Lett..

